# Hyperglycemia and Glycemic Variability Associated with Glucocorticoids in Women without Pre-Existing Diabetes Undergoing Neoadjuvant or Adjuvant Taxane Chemotherapy for Early-Stage Breast Cancer

**DOI:** 10.3390/jcm12051906

**Published:** 2023-02-28

**Authors:** Dana Mahin, Sayeh Moazami Lavasani, Leon Cristobal, Niki Tank Patel, Mina Sedrak, Daphne Stewart, James Waisman, Yuan Yuan, Wai Yu, Raynald Samoa, Nora Ruel, Susan E. Yost, Hayley Lee, Sung Hee Kil, Joanne E. Mortimer

**Affiliations:** 1 Department of Medical Oncology and Therapeutics Research, City of Hope Comprehensive Cancer Center, Duarte, CA 91010, USAjmortimer (J.E.M.); 2 Department of Clinical Diabetes, Endocrinology & Metabolism, City of Hope Comprehensive Cancer Center, Duarte, CA 91010, USA; 3 Department of Computational and Quantitative Medicine, City of Hope Comprehensive Cancer Center, Duarte, CA 91010, USA; 4 Arthur Riggs Diabetes & Metabolism Research Institute, City of Hope Comprehensive Cancer Center, Duarte, CA 91010, USA

**Keywords:** breast cancer, steroid-induced hyperglycemia, glycemic variability, glucocorticoids

## Abstract

Glucocorticoids, which are administered with chemotherapy, cause hyperglycemia. Glycemic variability among breast cancer patients without diabetes is not well known. A retrospective cohort study was conducted involving early-stage breast cancer patients without diabetes who received dexamethasone prior to neoadjuvant or adjuvant taxane chemotherapy between August 2017–December 2019. Random blood glucose levels were analyzed, and steroid-induced hyperglycemia (SIH) was defined as a random glucose level of >140 mg/dL. A multivariate proportional hazards model was used to identify the risk factors of SIH. Out of 100 patients, the median age was 53 years (IQR: 45–63.5). A total of 45% of patients were non-Hispanic White, 28% Hispanic, 19% Asian, and 5% African American. The incidence of SIH was 67%, and glycemic fluctuations were highest in those with glucose levels of >200 mg/dL. Non-Hispanic White patients represented a significant predictor for time to SIH, with a hazard ratio of 2.5 (95% CI: 1.04, 5.95, *p* = 0.039). SIH was transient in over 90% of the patients, and only seven patients remained hyperglycemic after glucocorticoid and chemotherapy completion. Pretaxane dexamethasone-induced hyperglycemia was observed in 67% of the patients, with the greatest glycemic lability in those patients with blood glucose levels of >200 mg/dL. The non-Hispanic White patients had a higher risk of developing SIH.

## 1. Introduction

Glucocorticoid treatment is ubiquitous across many diseases for curative treatment as well as supportive care. Glucocorticoids are frequently used prior to the administration of chemotherapy as antiemetics to minimize allergic reactions to the cremophor diluent with paclitaxel and to decrease third space fluid accumulation that is associated with docetaxel [1,2]. Notwithstanding their efficacy, glucocorticoids induce a wide range of side effects. Hyperglycemia is considered to have high clinical significance because it directly affects cancer treatment and has been reported to increase infection, organ dysfunction, and chemotherapy-associated toxicities [3,4,5,6]. It can also lead to chemotherapy dose delays or reductions [3,7], and decrease chemotherapy response [8].

Steroid-induced hyperglycemia (SIH) is an abnormal increase in blood glucose levels with the use of glucocorticoids [9] by reducing insulin sensitivity, increasing hepatic gluconeogenesis, and impairing beta cells and insulin secretion [9,10,11,12]. SIH is characterized by postprandial hyperglycemia, which is primarily mediated by insulin resistance. SIH is difficult to detect, challenging to prevent and treat, and vastly understudied. The clinical principle and practice of early detection, risk factor identification, and treatment algorithms do not exist for SIH. In addition, the prevalence of SIH is not known as studies use different criteria. Some studies use the steroid-induced diabetes diagnosis criteria set by the American Diabetes Association (ADA). although patients with random glucose levels > 200 mg/dL are asymptomatic and do not meet these criteria [10,13,14]. Other studies define SIH as any elevated glucose level outside of the normal range using fasting plasma glucose (FPG), oral glucose tolerance test (OGTT), and/or random blood glucose levels [9,10,14,15]. Given the transient nature of SIH with its resolution upon steroid discontinuation [13], SIH should be identified during glucocorticoid treatment as any abnormal elevation in glucose. Diabetes diagnosis should be made according to the ADA criteria and should be applied rigidly during, and if possible, after glucocorticoid treatment ends.

A meta-analysis in both nondiabetic and noncancer populations suggests that 32.3% of glucocorticoid-treated patients experience SIH [16]. It is a pervasive condition that can lead to complications, longer hospitalization, and mortality [17,18]. Since most studies are retrospective and no conclusive data are available from prospective randomized controlled clinical studies, the treatment targets and guidelines for asymptomatic and transient hyperglycemia have not been established [19]. In noncancer settings, SIH is associated with subsequent diabetes development, with the odds ratio ranging from 1.36 to 2.31 [20]. The risk factors for diabetes associated with SIH are not conclusive, but glucocorticoid-related parameters, such as dose, type, and duration of therapy, contribute more to diabetes development than traditional patient characteristics such as age, BMI, and family history [10]. 

Breast cancer is the most common cancer in the US, except for skin cancers [21]. Studies report widely different incidences of hyperglycemia associated with treatment ranging from 1% to 43%, but these data are not just limited to SIH and include clinical trials with novel therapies [22,23,24,25]. There is a paucity of research on the incidence, risk factors, prognosis, and outcomes of SIH in early-stage breast cancer patients undergoing adjuvant chemotherapy. The incidence of transient SIH during chemotherapy is virtually unknown in receptor subtypes of breast cancer, treatment type, and duration of treatment. Two recent studies investigated SIH prevalence in homogenous patient populations of Koreans; however, the applicability of disease course and risk factors may not be generalizable in other patient populations [4,14]. 

Owing to the uncertainties surrounding the incidence of SIH in early-stage breast cancer patients, we aim to determine the incidence of SIH and the degree of glycemic lability in women without pre-existing diabetes who received dexamethasone premedication for taxane-containing (neo) adjuvant chemotherapy. In this study, we sought to assess random blood glucose levels for each cycle of dexamethasone and taxane-containing chemotherapy and determine SIH prevalence and the degree of glycemic variability. We also aim to investigate the predictors of elevated glucose levels in early-stage breast cancer women.

## 2. Materials and Methods

A retrospective chart review was conducted at the City of Hope Comprehensive Cancer Center from 1 August 2017 to 31 December 2019. Women with breast cancer were considered eligible if they were >18 years of age, treated with paclitaxel or docetaxel in the neoadjuvant or adjuvant setting, received dexamethasone premedication, had no prior diagnosis of diabetes or prediabetes, and completed chemotherapy by 31 December 2019. Patients were excluded from the study if they had a diagnosis of diabetes before taxane chemotherapy or if they received chemotherapy outside the adjuvant or neoadjuvant setting (*n* = 100). The data collection included age, race/ethnicity, hypertension as a comorbidity, BMI, patient’s random blood glucose level throughout each cycle of chemotherapy, and treatment for hyperglycemia after completion of therapy. 

Patients who had hyperglycemia defined by random blood glucose > 140 mg/dL after receiving steroid treatment were recorded. Glycemic thresholds do not exist for SIH other than traditional glucose concentrations used for prediabetes and diabetes diagnosis [13]. In this study, we defined the higher-level SIH group as >200 mg/dL, the lower-level SIH group as 140–199 mg/dL, and the euglycemia group as <140 mg/dL. When accompanied by hyperglycemic symptoms, these thresholds for random glucose levels indicate a definitive diagnosis of prediabetes and diabetes per ADA. In this study, random rather than fasting glucose was used because glucocorticoids generally cause exaggerated postprandial hyperglycemia and have a lesser effect on fasting glucose. HbA1C is typically not evaluated for breast cancer patients undergoing therapy. Thus, no other metrics from blood tests were used.

The baseline random blood glucose was reported 1–4 weeks prior to the start of chemotherapy. The random blood glucose readings after initiation of chemotherapy were taken on the first day of each chemotherapy cycle after the required dose of dexamethasone premedication was given. The follow-up random blood glucose was reported 3–4 weeks after all cycles of chemotherapy were concluded. In addition, follow-up glucose levels were monitored after 2–3 months, 6 months, 1 year, and 2 years.

Data were summarized using median and interquartile range (IQR) for continuous variables and frequency/percentage for categorical fields. Data distribution was compared across categories of patients for euglycemia (<140 mg/dL), lower-level SIH (140–199 mg/dL), and higher-level SIH (>200 mg/dL) using the Wilcoxon rank sum test or t-test for continuous and chi-square or fisher’s exact test for categorical data, as deemed appropriate. Time to first elevated glucose level at or above 200 mg/dL was calculated, and Kaplan-Meier was used to estimate the probability of event-free survival. Univariate and multivariate proportional hazards models were used to test age, race/ethnicity, BMI, baseline glucose level, hypertension, and type of taxane as predictors for time to first elevated blood glucose level at or over 200 mg/dL. Graphs were generated to depict changes in blood glucose from baseline, using the direct ratio of values after initiation of treatment to the baseline value for each patient. The threshold for significance was set at 0.05. SAS^®^ 9.4 was used to conduct data analysis and generate figures.

## 3. Results

A total of 131 female patients with breast cancer were identified. Thirty-one patients were excluded due to metastatic cancer (n = 16), type 2 diabetes (n = 13), and blood glucose levels at or higher than 200 mg/dL prior to the initiation of chemotherapy (n = 2). Table 1 summarizes the baseline patient characteristics for the 100 patients enrolled in the study. The random glucose assessment identified 45 (45%) patients with lower-level SIH, 22 (22%) with higher-level SIH, and 33 (33%) with euglycemia. The median age was 53 years with an interquartile range (IQR) of 45–63.5 years, with 45% non-Hispanic White, 28% Hispanic, 19% Asian, 5% African American, and 3% unknown. The median BMI was 27.1 kg/m^2^ (IQR, 23.7–30.8). According to BMI classification [26], 1% was underweight (BMI < 18.5), 32% were normal weight (BMI 18.5 to <25), 40% were overweight (BMI 25 to <30), 16% were obese (BMI > 30), and 11% were morbidly obese (BMI > 40). Twenty-four percent of patients were hypertensive, and 63% gained weight during treatment which consisted of docetaxel (51%) and paclitaxel (49%). A total of 67% of patients were estrogen receptor (ER) positive, 48% were progesterone receptor (PR) positive, and 38% were human epidermal growth factor receptor 2 (HER2) positive. 

The baseline random blood glucose range was 68–186 mg/dL. Patients with a blood glucose level of >200 mg/dL were excluded from the study, and three patients with glucose levels of 140–199 mg/dL were included in the analysis. The pretreatment glucose levels were available for 84 patients with a median glucose level of 99 mg/dL (IQR, 91.5–109.5). Out of the 16 patients without pretreatment glucose levels, 11 had elevated glucose levels (140–199 mg/dL range), and two had >200 mg/dL post dexamethasone treatment (Table 2). 

The median age for the 22 patients with at least one blood glucose level of > 200 mg/dL was 59.5 years (range 50–68). Out of these patients, 14 are non-Hispanic White (63.6%), 7 are Hispanic (31.8%), and 1 is Asian (4.5%) (Table 1). Seven (31.8%) patients were obese or morbidly obese, and 8 (36.4%) were overweight. Eight (36.4%) patients were on hypertensive therapy. Patients received a median of 4 cycles of chemotherapy, and 11 (50%) patients had glucose levels of >200 mg/dL during the first cycle, while 5 (22.7%) patients reached >200 mg/dL during the second cycle and 6 (27.3%) patients between the 3rd and 6th cycles.

The median age of the 45 patients in the lower-level SIH group was 53 years (range 45–64), 19 (42.2%) were overweight, and 11 (24.5%) were obese or morbidly obese. Eighteen patients (40%) are non-Hispanic White, 11 (24.4%) are Hispanic, 11 (24.4%) are Asian, 2 (4.4%) are African American, and 3 (6.7%) are unknown. Nine (20.0%) patients were on antihypertensive therapy. These patients received a median of four cycles of chemotherapy. 

Out of the 33 patients who consistently maintained a blood glucose level less than 140 mg/dL, their median age was 51 years (range 43–59). Thirteen (39.4%) were overweight, and nine (27.3%) were obese or morbidly obese. Thirteen (39.4%) are non-Hispanic White, 10 (30.3%) are Hispanic, 7 (21.2%) are Asian, and 3 (9.1%) are African American. Seven (21.2%) patients were being treated for hypertension. A median of five cycles of chemotherapy was administered among the euglycemia patients.

**Figure 1:**
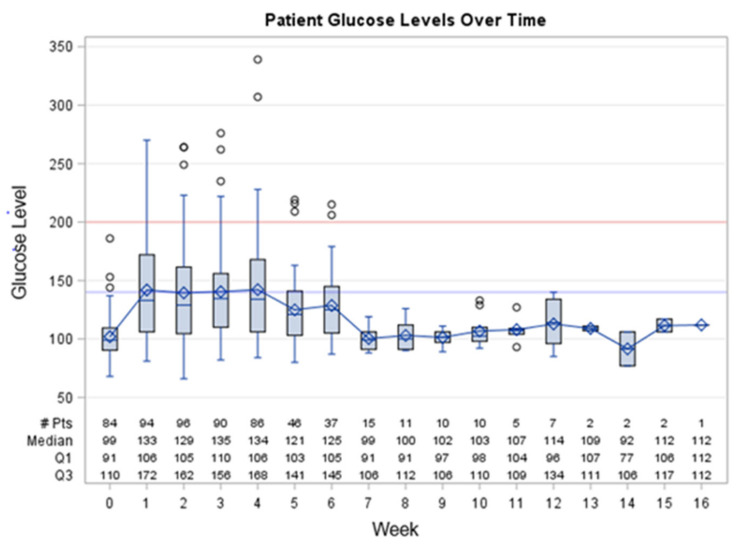
Median blood glucose levels from dexamethasone initiation at week 0 to treatment completion at week 16 (chemotherapy cycle 1 through cycle 4). Overall changes in blood glucose for all patients are reported by cycle and are expressed as the median and IQR, including outliers. Values inside box range from lower to upper quartile; whiskers are drawn from the box to the most extreme point that is ≤1.5 × IQR; observations outside this range are plotted individually. Glucose level of 200 is indicative of diabetes. IQR: interquartile range; # pts, number of patients.

Figure 1 shows the median blood glucose level from dexamethasone initiation at week 0 to treatment completion at week 16, which corresponds to the duration of chemotherapy cycle 1 through to cycle 4. The elevations in glucose levels were seen as early as the first week and remained high until week 6. Most reverted to the normal range around week 7 and remained normoglycemic until the end of chemotherapy.

**Figure 2:**
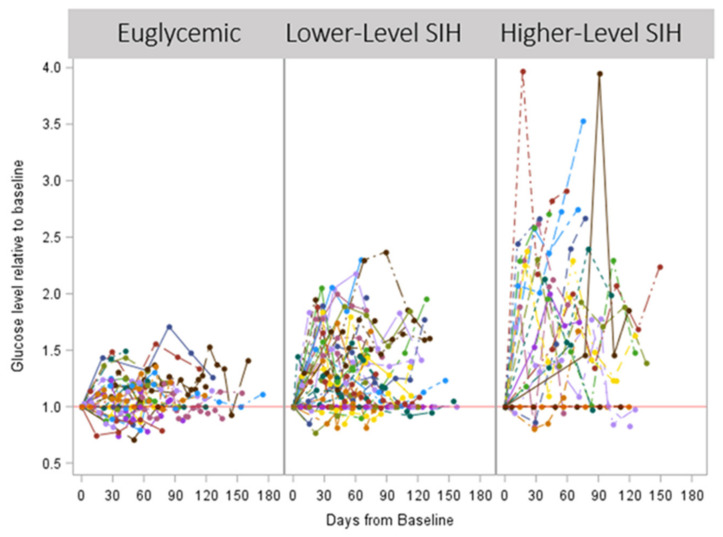
Fluctuations in glucose levels relative to baseline are plotted for euglycemic patients, lower-level SIH patients, and higher-level SIH patients. Spider plot shows the glucose changes for each patient over the course of chemotherapy, with the greatest fluctuations observed in the higher-level SIH patients. Reference line 1 indicates baseline value.

In Figure 2, the fluctuations in glucose levels over time are plotted for the euglycemic patients, lower-level SIH patients, and higher-level SIH patients. The greatest fluctuations were observed in the higher-level SIH patients.

**Figure 3:**
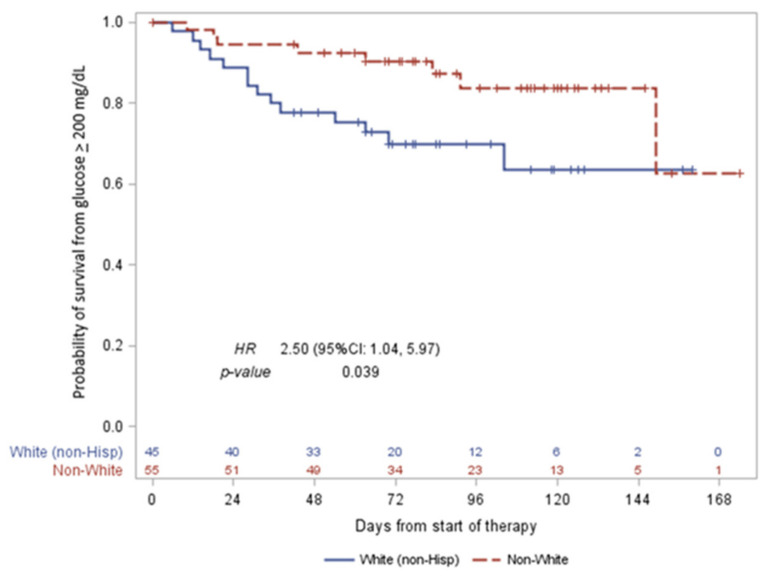
Kaplan–Meier univariate proportional hazard model between non-Hispanic White (non-Hispanic) and non-White patients for time to elevated blood glucose > 200 mg/dL. Race/ethnicity showed a statistical significance in the univariate model.

Univariate proportional hazards models were used to test whether age (<50 vs. ≥50), race/ethnicity (non-Hispanic White vs. non-White), BMI (≤normal BMI vs. obese/morbidly obese), baseline glucose level (continuous), hypertension (yes vs. no), and taxane type (docetaxel vs. paclitaxel) were significant predictors of time to first elevated blood glucose level > 200 mg/dL. Race/ethnicity was the only significant predictor, with a hazard ratio (HR) of 2.5 (95% CI: 1.04, 5.95, *p* = 0.039) in the White vs. non-White patients. Figure 3 shows a Kaplan–Meier curve for time to elevated blood glucose > 200 mg/dL in the White vs. non-White patients. Race/ethnicity showed a statistical significance in the univariate model; however, the multivariate model did not maintain this significance.

Postchemotherapy random blood glucose levels were available for 96 patients, and of these, 89/out of 96 (93%) patients had glucose levels within the normal range. Five had glucose levels between 140 and 199 mg/dL, and two had glucose levels at or over 200 mg/dL. None of the patients received treatment for hyperglycemia or were referred for an endocrinology consultation. More extensive follow-up revealed that after 2–3 months, four patients had hyperglycemia. At 6 months follow-up, seven patients had hyperglycemia (one with >200). At 1-year follow-up, 16 patients had hyperglycemia (one with >200), and at 2-year follow-up, 11 patients had hyperglycemia (two with >200).

## 4. Discussion

This study sought to identify the incidence of steroid-induced hyperglycemia and the degree of glycemic variability among early-stage breast cancer patients undergoing chemotherapy. A total of 67 women (67%) without a known diagnosis of diabetes or prediabetes had hyperglycemia during (neo) adjuvant chemotherapy using dexamethasone and either docetaxel or paclitaxel. Given the intermittent use of glucocorticoids prior to chemotherapy and no continuous or extended administration, the incidence of hyperglycemia was high. Recent studies in a similar clinical context in breast cancer patients undergoing adjuvant chemotherapy indicate a much lower incidence of SIH, with one study reporting 3.3% (5/152) [4] and another reporting 19% (82/423) [14]. The threshold of SIH was defined differently, where the former included patients whose random glucose was >200 mg/dL, whereas the latter included random glucose > 140 mg/dL. In previous studies involving breast cancer patients that investigated hyperglycemia across all cancer stages with various treatments, including new pharmacological agents and glucocorticoids, the incidence of hyperglycemic events was reported to be 1–43% [22,23,24,25,27].

Our observed high incidence of SIH (67%) was even higher than other cancer patient types whose treatment included a high glucocorticoid dose and extended treatment duration. Hyperglycemia among cancer patients with brain tumor/metastasis, lymphoma, or bone marrow transplant (BMT) is reported to be 58.9% [28]. Other cancer types with reported hyperglycemia include acute lymphocytic leukemia (58.1%) [29], non-Hodgkin lymphoma (43.2%), and prostate cancer (49.2%) [3]. Of note, these studies are of largely hospitalized cancer patients who either underwent bone marrow transplants and/or metastatic cancer patients on continuous high steroid use for a longer duration. The current study included early-stage breast cancer patients who received intermittent glucocorticoids as a pretreatment regimen for chemotherapy in an outpatient setting.

After completion of dexamethasone and chemotherapy, 93% of patients were found to have random glucose levels that were within the normal range. In previous studies involving breast cancer patients, 6 out of 17 (35.3%) patients with SIH remained in a hyperglycemic state after chemotherapy [4]. In another study, 10 out of 82 (12.2%) progressed to newly diagnosed diabetes [14]. We did not measure or investigate other diagnostic criteria for diabetes, such as HbA1c, oral glucose tolerance test (OGTT), or fasting plasma glucose (FPG).

Although SIH is transient and many patients revert to a euglycemic state, the data indicate that even transient hyperglycemic episodes are associated with decreased mortality and complications among the noncancer patient population [30,31,32]. The relationship between the observed high prevalence of SIH in our patient population and clinical outcomes has not been analyzed in this study. More analyses are needed to fully elucidate the impact of hyperglycemia during chemotherapy on subsequent diabetes occurrence, cancer prognosis, and cancer outcomes, including mortality. A recent study suggests that SIH in breast cancer patients may be associated with lower relapse-free survival, but more studies are warranted [14].

We found a significantly higher incidence of glucose dysregulation in non-Hispanic White patients compared with other races/ethnicities. To date, this is the first study to evaluate the prevalence of SIH across different race/ethnicities among early-stage breast cancer patients. Diabetes and diabetes-related complications disproportionately affect racial and ethnic minority groups. Hispanic and African American patients have one of the highest rates, and non-Hispanic White patients have the lowest rate [33]. Interestingly, the frequency of hyperglycemia that we observed in this study suggests a different racial/ethnicity distribution, with non-Hispanic White patients having the highest SIH rate.

In terms of glucose lability in our patient population, the degree of glucose excursions was greatest in the higher-level SIH group compared to lower-level SIH and euglycemic groups. Glycemic excursions are dependent on the pharmacokinetic and pharmacodynamic properties of glucocorticoids as well as the severity of decreased insulin sensitivity. In general, blood glucose levels are elevated postprandially in the latter part of the day, with stabilized levels observed in the morning [10,11,19]. The duration of glucose variability can be short-term or long-term, with fluctuations both within-day and day-to-day. Both short and long-term glucose variability is associated with tissue damage, microvascular complications, cardiovascular diseases, and mortality in the noncancer patient population [34,35]. Studies of cancer patients are needed to assess the relationship between glucose fluctuations and clinical outcomes. The deleterious effects of glucose variability are driven by oxidative stress, endothelial dysfunction, and inflammation [34]. Hyperglycemia increases the reactive oxygen species production and inactivates nitric oxide, which results in endothelial dysfunction and vascular complications [36,37]. In addition to hyperglycemia, glucose lability is associated with changes in endothelial nitric oxide synthase, increased oxidate stress, and tissue damage via intracellular signal transduction pathways, including protein kinase B (AKT) [34,38]. Of note, glucose variability is associated with microvascular complications, cardiovascular outcomes, and mortality independent of HbA1c [39,40]. 

For patients without diabetes, SIH is linked to the subsequent development of diabetes, with the odds ratio for diabetes reported to be 1.36–2.31 [20]. Diabetes increases chemotherapy toxicities and complications [41], poor breast cancer prognosis [42,43], diabetes-related mortality [44], cardiovascular mortality [44], breast cancer-specific mortality [44], and overall mortality [41,42,43,45] in breast cancer patients. However, SIH is difficult to monitor in cancer patients because patients are either asymptomatic or have symptoms such as dry mouth, fatigue, and polyuria, which may be attributed to cancer therapy. In addition, the diagnosis of diabetes after chemotherapy and surgery may be delayed or go undetected due to a lack of continuity of care in post-treatment survivors. 

Our study demonstrated the high prevalence of SIH and glycemic variability among early breast cancer patients undergoing (neo) adjuvant chemotherapy, specifically for non-Hispanic White patients. Though most patients revert to euglycemia, more definitive diagnostic tests such as HbA1c, OGTT, and FPG are needed to accurately diagnose patients who progress to newly developed diabetes post-SIH. This is of high clinical significance as diabetes in breast cancer patients is associated with worse prognosis, complications, and mortality. SIH patients, specifically those whose blood glucose level is > 200 mg/dL and patients with a high degree of glucose excursions, should be monitored closely and should be considered for an endocrinology consultation for further workup. Moreover, these patients may be considered for continuous glucose monitoring (CGM), which can automatically and serially track glucose levels, or self-monitoring of blood glucose (SMBG) using glucometers to monitor glucose variability during and after chemotherapy. CGM and SMBG might be especially important in this patient population as the dose, frequency, and duration of steroid use vary across patients, and the increases and fluctuations in glucose levels may not be detected during routine laboratory assessments. Given that steroids primarily increase postprandial blood glucose levels, the diagnostic sensitivity of SIH is greatest in the afternoon or in the evening, but most patients get tested in the morning during their clinic visits. CGM and SMBG may capture SIH that are otherwise underdiagnosed and underreported due to this limitation. 

The study has several limitations, including a small sample size and limiting the study to patients without prediabetes or diabetes. The incidence and glucose lability among patients with diabetes was not investigated. Information on the history of gestational diabetes or family history of diabetes was not collected. Although random glucose levels were extracted at the end of chemotherapy to determine the persistence of SIH, these were not definitive diagnostic tests for diabetes, and the follow-up data after chemotherapy were not assessed. Notwithstanding these limitations, our study, to our knowledge, is the first study to examine the incidence of SIH across different races/ethnicities in early breast cancer patients undergoing (neo) adjuvant chemotherapy prior to surgery. Though additional follow-up is needed to investigate the incidence of subsequent diabetes development, the study revealed that SIH is prevalent, with a high degree of glycemic variability, particularly in non-Hispanic White patients. Close monitoring with CGM and SMBG may be needed for patients at risk of hyperglycemia. Diagnostic tests for diabetes, such as HbA1c, OGTT, FPG, and endocrinology consultation, should also be recommended. Moreover, further research is warranted to examine whether transient SIH is associated with cancer prognosis, complications, and survival.

## Figures and Tables

**Table 1:** Characteristics of patients (n = 100) classified as higher-level SIH (glucose > 200 mg/dL), lower-level SIH (glucose 140–199 mg/dL), and euglycemia (<140 mg/dL) ^1^.

	Euglycemia (n = 33)	Lower-Level SIH (n = 45)	Higher-Level SIH (n = 22)	*p*-Value	Total (n = 100)
Race					
Asian/Pacific Islander	7 (21.2%)	11 (24.4%)	1 (4.5%)	0.2	19
Black or African American	3 (9.1%)	2 (4.4%)	0 (0.0%)		5
Hispanic or Latino	10 (30.3%)	11 (24.4%)	7 (31.8%)		28
White	13 (39.4%)	18 (40.0%)	14 (63.6%)		45
Other/Unknown	0 (0.0%)	3 (6.7%)	0 (0.0%)		3
BMI	27.7 (23.8, 30.3)	27.0 (23.4, 29.9)	28.1 (23.7, 31.9)	0.6	27.1 (23.7, 30.8)
BMI Group					
Underweight	1 (3.0%)	0 (0.0%)	0 (0.0%)	0.8	1 (1%)
Normal	10 (30.3%)	15 (33.3%)	7 (31.8%)		32 (32%)
Overweight	13 (39.4%)	19 (42.2%)	8 (36.4%)		40 (40%)
Obese	4 (12.1%)	8 (17.8%)	4 (18.2%)		16 (16%)
Morbidly obese	5 (15.2%)	3 (6.7%)	3 (13.6%)		11 (11%)
Age	51 (43, 59)	53 (45, 64)	59.5 (50, 68)	0.04	53 (45, 63.5)
Hypertension					
No	26 (78.8%)	36 (80.0%)	14 (63.6%)	0.3	76
Yes	7 (21.2%)	9 (20.0%)	8 (36.4%)		24
Baseline Glucose	99.5 (89–110)	98.5 (90–100)	99.5 (95, 113)	0.9	99 (90.5–109.5)
Max Weight Gain (over all cycles)					
Number who gained >0 kg	20 (60.6%)	28 (62.2%)	15 (68.2%)	0.8	63
Maximum weight gained (kg)	1.2 (0.9, 2.5)	1.6 (1.0, 2.8)	1.4 (0.6, 3.8)	0.9	1.4 (0.9, 2.6)
Treatment					
Docetaxel	13 (39.4%)	26 (57.8%)	12 (54.5%)	0.3	51
Paclitaxel	20 (60.6%)	19 (42.2%)	10 (45.5%)		49
ER					
Negative	15 (45.5%)	13 (28.9%)	5 (22.7%)	0.3	33
Positive	18 (54.5%)	32 (71.1%)	17 (77.3%)		67
PR					
Negative	22 (66.7%)	21 (46.7%)	9 (40.9%)	0.2	52
Positive	11 (33.3%)	24 (53.3%)	13 (59.1%)		48
HER2					
Negative	21 (63.6%)	29 (64.4%)	12 (54.5%)	0.9	62
Positive	12 (36.4%)	16 (35.6%)	10 (45.5%)		38

^1^ N (%) or Median; (IQR: interquartile range; BMI: body mass index; ER: Estrogen receptor; PR: progesterone receptor; HER2: human epidermal growth factor receptor 2; SIH: steroid-induced hyperglycemia; HER2 positivity defined as IHC score 2–3+, gene amplification on FISH > 2.0 (>10% tumor cells).

**Table 2:** Number of patients with baseline blood glucose levels observed in euglycemia, lower-level SIH, and higher-level SIH.

Random Blood Glucose mg/dL	Euglycemia (n = 33)	Lower-Level SIH (n = 45)	Higher-Level SIH (n = 22)	Total
Median baseline glucose level (IQR)	99.5 (89–110)	98.5 (90–105)	99.5 (95–113)	99 (90.5–109.5)
Normal (<140)	30 (90.9%)	32 (71.1%)	19 (86.4%)	81
Pre-diabetes range (140–199)	0 (0.0%)	2 (4.4%)	1 (4.6%)	3
No baseline glucose	3 (9.1%)	11 (24.4%)	2 (9.1%)	16

IQR: interquartile range; SIH: steroid-induced hyperglycemia.

## Data Availability

The data presented in this study are available on request from the corresponding author. The data are not publicly available due to privacy or ethical issues.
